# Combination of a Latency-Reversing Agent With a Smac Mimetic Minimizes Secondary HIV-1 Infection *in vitro*

**DOI:** 10.3389/fmicb.2018.02022

**Published:** 2018-09-19

**Authors:** Shin-ichiro Hattori, Kouki Matsuda, Kiyoto Tsuchiya, Hiroyuki Gatanaga, Shinichi Oka, Kazuhisa Yoshimura, Hiroaki Mitsuya, Kenji Maeda

**Affiliations:** ^1^National Center for Global Health and Medicine Research Institute, Tokyo, Japan; ^2^AIDS Clinical Center, National Center for Global Health and Medicine, Tokyo, Japan; ^3^AIDS Research Center, National Institute of Infectious Diseases, Tokyo, Japan; ^4^Experimental Retrovirology Section, HIV and AIDS Malignancy Branch, National Cancer Institute, National Institutes of Health, Bethesda, MD, United States

**Keywords:** HIV, latency-reversing agent, Smac mimetic, birinapant, PKC activator, caspase-3

## Abstract

Latency-reversing agents (LRAs) are considered a potential tool to cure human immunodeficiency virus type 1 (HIV-1) infection, but when they are taken alone, virus production by reactivated cells and subsequent infection will occur. Hence, it is crucial to simultaneously take appropriate measures to prevent such secondary HIV-1 infection. In this regard, a strategy to minimize the production of infectious viruses from LRA-reactivated cells is worth pursuing. Here, we focused on a second mitochondria-derived activator of caspases (Smac) mimetic, birinapant, to induce apoptosis in latent HIV-1-infected cells. When birinapant was administered alone, it only slightly increased the expression of caspase-3. However, in combination with an LRA (e.g., PEP005), it strongly induced the expression of caspase-3 followed by enhanced apoptosis. Importantly, the combination eliminated reactivated cells and drastically reduced HIV-1 production. Finally, we found that birinapant decreased the mRNA expression of HIV-1 that was induced by PEP005 in the primary CD4^+^ T-cells from HIV-1-carrying patients as well. These results suggest that the combination of an LRA and an “apoptosis-inducing” agent, such as a Smac mimetic, is a possible treatment option to decrease HIV-1 reservoirs without the occurrence of HIV-1 production by reactivated cells.

## Introduction

Despite prolonged antiretroviral therapy (ART), human immunodeficiency virus type 1 (HIV-1) persists in the virus reservoir, and in this regard, the use of latency-reversing agents (LRAs) is considered as a potential strategy to cure HIV-1 infection. Several small molecules have been reported as LRAs. They reactivate latent HIV-1-infected cells and increase HIV-1 RNA expression. After reactivation, the latent cells are subsequently killed by cytotoxic T lymphocytes (CTL) or viral cytopathic effects (Choudhary and Margolis, 2011; Karn, 2011; Hakre et al., 2012; Bullen et al., 2014).

Several candidates have recently been reported as LRAs, including protein kinase C (PKC) agonists (activators) (e.g., PEP005 and bryostatin-1), histone deacetylase (HDAC) inhibitors (e.g., SAHA/Vorinostat), and BRD4 inhibitors (e.g., JQ1) (Warrilow et al., 2006; Bullen et al., 2014; Cillo et al., 2014; Spivak et al., 2015; Cillo and Mellors, 2016). Recent studies have shown that it is important for such drugs to be used in combination to obtain higher levels of HIV-1-RNA transcription as multiple mechanisms are involved in maintaining the transcriptionally silent state of HIV-1 (Donahue and Wainberg, 2013; Mbonye and Karn, 2014). However, there are still many obstacles in using LRAs to disrupt HIV latency *in vivo*. Several LRAs have been tested in clinical trials with varying degrees of success; some studies have demonstrated that the size of the latent reservoir remained unchanged, indicating that these drugs failed to purge the virus *in vivo* (Archin et al., 2012; Rasmussen et al., 2014; Elliott et al., 2015; Gutiérrez et al., 2016). These results also imply that *in vitro*/*ex vivo* LRA potency does not necessarily translate into clinical LRA potency. Moreover, hypothetically, if LRAs are used alone in treatment-naïve HIV patients, infectious viruses will be produced from reactivated reservoir cells and subsequent infection of uninfected cells will occur. Thus, for effective LRA therapy against HIV-1 reservoirs, it is essential to use additional potent antiretroviral drugs to prevent new infection. One proposal is to combine LRAs with existing anti-HIV drugs, such as reverse transcriptase (RT) inhibitors, protease inhibitors, and integrase inhibitors. Furthermore, the combined use of anti-HIV-1 drugs with LRAs is also regarded as a valuable strategy to minimize production/secretion of infectious viruses by reactivated cells. Recently, Tateishi et al. (2017) proposed a new strategy where the infecting virus is “locked-in” the host cells, and this is followed by the process of apoptosis in the presence of a newly reported compound (L-HIPPO), suggesting that such drugs may decrease the levels of latent HIV-1-infected cells without producing infective viruses (Tateishi et al., 2017).

The tumor necrosis factor (TNF) receptor 1 complex receives TNF signals and then transmits them inside the cell, triggering a cascade that includes the activation of nuclear factor (NF)-κB, which leads to the reversal of HIV-1 latency. Alternatively, second mitochondria-derived activator of caspases (Smac) mimetics, which are known to inhibit TNF and induce NF-κB activation, have been developed as therapeutics for multiple cancers. They are considered as a new class of cancer therapeutics that is well tolerated *in vivo*. Primarily through their antagonism of cellular inhibitor of apoptosis (cIAP), they promote apoptosis in tumor cells, while normal tissue remains unaffected (Fulda and Vucic, 2012; Bai et al., 2014; Pache et al., 2015). Birinapant is one such Smac mimetic that was also developed as a therapeutic agent against cancer (Allensworth et al., 2013; Krepler et al., 2013; Bai et al., 2014). However, Ebert et al. (2015) reported that birinapant and other Smac mimetics also rapidly reduced the serum hepatitis B virus (HBV) DNA levels and the serum HBV antigens (Ebert et al., 2015). The authors demonstrated that a single administration of birinapant resulted in a decrease in the number of HBV core antigen (HBcAg)-expressing hepatocytes in a mice model. In addition, this decrease was associated with an increase in the number of terminal dUTP nick end labeling (TUNEL)-positive cells (Ebert et al., 2015), suggesting that the administration of birinapant successfully decreased the number of HBV-positive cells via apoptosis.

In this study, we focused on the effect of apoptosis on latent HIV-1-infected cells when treated with a Smac mimetic, especially when combined with LRA. We found that the combination of birinapant and PEP005 (ingenol-3-angelate, PKC activator) induced the strong upregulation of caspase-3 followed by enhanced apoptosis of latent HIV-1-infected cells.

## Materials and Methods

### Drugs and Reagents

Birinapant (IAP inhibitor) and GS-9620 (TLR-7 agonist) were purchased from MedChem Express (Monmouth Junction, NJ, United States). PEP005 (PKC activator), SAHA (Vorinostat, HDAC inhibitor), JQ-1 (BRD4 inhibitor), GSK525762A (BRD4 inhibitor), Ro5-3335 (CBFβ/RUNX inhibitor), and Al-10-49 (CBFβ/RUNX inhibitor) were purchased from Cayman Chemical (Ann Arbor, MI, United States), Santa Cruz Biotechnology (Dallas, TX, United States), BioVision (Milpitas, CA, United States), ChemScene (Monmouth Junction, NJ, United States), Merck (Darmstadt, Germany), and Selleck (Houston, TX, United States), respectively. Phorbol myristate acetate (PMA) and TNF-α were purchased from Wako Pure Chemical (Osaka, Japan) and BioLegend (San Diego, CA, United States), respectively.

### Cells and Viruses

Latent HIV-1-infected model cell line U1 (Folks et al., 1988) derived from the U937 promonocytic cell line and ACH-2 cells (Clouse et al., 1989) derived from the A3.01 T lymphocytoid cell line were used for this study. J1.1 cell line (Perez et al., 1991) derived from Jurkat cells were also used. These cell lines were obtained from the National Institutes of Health (NIH) AIDS Reagent Program. Cells were maintained in RPMI 1640 medium (Sigma-Aldrich, St. Louis, MO, United States) supplemented with 10% fetal calf bovine serum (FCS, Sigma-Aldrich), 50 U/μl penicillin, and 50 μg/ml kanamycin.

### Antiviral Assays and Cytotoxicity Assays

Antiviral assays using wild type HIV-1 (HIV-1 NL4-3) cells and MT-4 cells were conducted as previously described (Maeda et al., 2014; Takamatsu et al., 2015). To determine the cytotoxicity induced by LRAs and its related compounds, cells (5 × 10^5^ cells/mL) were cultured in the presence or absence of LRA. After 24 h, cytotoxicity assays were performed using a Cytotoxicity LDH Assay Kit (Dojindo, Kumamoto, Japan) according to the manufacturer’s instructions. The number of living cells with the drug was measured and compared with those without the drug, and data are shown as percentage (%) of control.

### HIV Reactivation From Latent HIV-1-Infected Cells

The reactivation of HIV-1 from latently infected cells was determined by the measurement of supernatant p24 antigen and intracellular HIV-1 RNA levels. ACH-2 cells or U1 cells (5 × 10^5^ cells/mL) were placed in 96-well plates, and then the cells were incubated with different concentrations of drugs for 24 h to extract RNA and for 48 h to collect the supernatant. The increase in supernatant p24 antigen levels was measured using LUMIPULSE G1200 (Fujirebio, Tokyo, Japan). The total RNA was extracted using an RNeasy Mini Kit (Qiagen, Hilden, Germany), following the manufacturer’s protocols. The cDNA was synthesized using PrimeScript RT Master Mix (Takara Bio, Shiga, Japan), and quantitative real-time PCR analyses for the intracellular HIV-1 RNA were carried out with PowerUp^TM^ SYBR Green Master Mix (Applied Biosystems, Foster City, CA, United States). The oligonucleotide primers used were as follows: 5′-TGTGTGCCCGTCTGTTGTGT-3′ (forward) and 5′-GAGTCCTGCGTCGAGAGAGC-3′ (reverse) for HIV-RNA detection; 5′-GCGAGAAGATGACCCAGATC-3′ (forward) and 5′-CCAGTGGTACGGCCAGAGG-3′ (reverse) for β-actin. The HIV-RNA expressions were normalized with that of β-actin, which was indicated as the relative quantity, and the increase in HIV-1 RNA levels in the presence of a drug was compared with that without a drug.

### Primary CD4^+^ T-Cells Isolation From HIV-1 Patient Samples and HIV-1 Reactivation Using Drugs

Peripheral blood samples were collected from HIV-1-infected patients who had been receiving combination antiretroviral therapy (cART) for at least 6 years. All subjects maintained lower viral load (<20 copies/ml) (except for occasional blips) during therapy. CD4^+^ T-cell counts in peripheral blood samples ranged from 464–807 cells/mm^3^ (average 601 cells/mm^3^), and plasma viral loads were <41.3 copies/ml as measured by qPCR (Roche COBAS AmpliPrep/COBAS TaqMan HIV-1 Test version 2.0) at the time of study enrollment. Written informed consent was obtained from all the subjects. The Ethics Committee at the National Center for Global Health and Medicine approved this study (NCGM-G-002259-00), and each patient provided a written informed consent. Whole peripheral blood mononuclear cells (PBMCs) were separated by density gradient centrifugation with Ficoll-Paque^TM^ (GE Healthcare, Munich, Germany), and CD4^+^ T-cells were purified using MojoSort^TM^ Human CD4^+^ T Cell Isolation Kit (BioLegend, San Diego, CA, United States) according to the manufacturer’s instructions. The purified CD4^+^ T-cells were plated at a density of 2.5 × 10^6^ cells/ml and were treated with 100 nM PMA plus 2 μM Ionomycin, 50 nM Birinapant, and/or 5 nM PEP005 for 24 h, and the cells were collected for RNA purification. The total RNA was extracted, cDNA was synthesized, and quantitative real-time PCR analyses for studying the expression of intracellular HIV-1 RNA were carried out as shown above. The HIV RNA copies were calculated using a standard curve obtained from serially diluted HIV-1 plasmid, the normalized values [HIV RNA copies/input RNA (ng)] with a drug were compared with that without a drug, and the relative increase in HIV-1 RNA levels in the presence of a drug was determined.

### FACS Analysis

Intracellular HIV-1 p24 levels and active form of caspase-3 expression were determined as previously described (Hattori et al., 2009; Duro et al., 2010; Matsuda et al., 2015). In brief, ACH-2 cells or U1 cells (2.5 × 10^5^ cells/mL) were fixed with 1% paraformaldehyde/phosphate buffered saline (PBS) for 20 min and were permeabilized with Flow Cytometry Perm Buffer (TONBO biosciences, San Diego, CA, United States). After incubation on ice for 5 min, the cells were stained with anti-HIV-1 p24 (24-4)-FITC mAb (Santa Cruz Biotechnology, Dallas, TX, United States) and/or with Alexa Fluor 647-conjugated anti-active caspase-3 mAb (C92-605) (BD Pharmingen, San Diego, CA, United States) for 30 min on ice. Cells were then analyzed on a BD FACSVerse flow cytometer (BD Biosciences, Franklin Lakes, NJ, United States). The corrected data were analyzed with FlowJo software (Tree Star, San Carlos, CA, United States).

### Determination of cIAP Expression

ACH-2 cells (1 × 10^6^ cells/ml) were treated with different concentrations of the drug for 24 h, and the cells were washed twice with PBS. Cells were homogenized by CelLytic M Cell Lysis Reagent (Sigma-Aldrich, St. Louis, MO, United States) containing a protease inhibitor cocktail (Nacalai Tesque, Kyoto, Japan) and were incubated at room temperature (RT) for 15 min. The cells were then centrifuged at 13,000 rpm for 15 min, and the supernatant of the whole cell lysate was collected. The bicinchoninic acid (BCA) protein assay (Thermo Scientific, Rockford, IL, United States) was used to measure the actual protein amount in the treated and untreated cells. The protein extracts were separated through sodium dodecyl sulfate polyacrylamide gel electrophoresis (SDS-PAGE) and then were blotted onto a Trans Blot Turbo polyvinylidene difluoride (PVDF) membrane (Bio-Rad, Hercules, CA, United States). Protein bands were visualized using 3,3′,5,5′-tetramethylbenzidine (TMB) solution (Wako Pure Chemical). The sources of the antibodies were as follows: c-IAP1 (D5G9), c-IAP2 (58C7), and XIAP (3B6) were purchased from Cell Signaling Technology (Danvers, MA, United States); β-actin (C4) was purchased from Santa Cruz Biotechnology.

### Determination of TNF-α mRNA Expression and Production in Culture Supernatants

The expression of TNF-α mRNA was measured as previously described (Zhang et al., 2014). For the detection of supernatant TNF-α levels, ACH-2 cells (2.5 × 10^5^ cells/ml) were incubated with various concentrations of PEP005 (0–50 nM) for 24 h at 37°C. Culture supernatants were harvested and centrifuged to remove cellular debris. The production of TNF-α was measured by using a Human TNF alpha Platinum ELISA Kit (eBioscience, Hatfield, United Kingdom) according to the manufacturer’s instructions. The sensitivity of the assay was 2.3 pg/ml.

### Statistical Analysis

Differences between groups were analyzed for statistical significance using the Mann-Whitney U test. *P*-values < 0.05 denoted the presence of statistically significant difference. Analysis was performed using the GraphPad Prism software version 4 (La Jolla, CA, United States).

## Results

### LRAs Reactivate and Induce HIV-1 Production in Latent Cells

**FIGURE 1:**
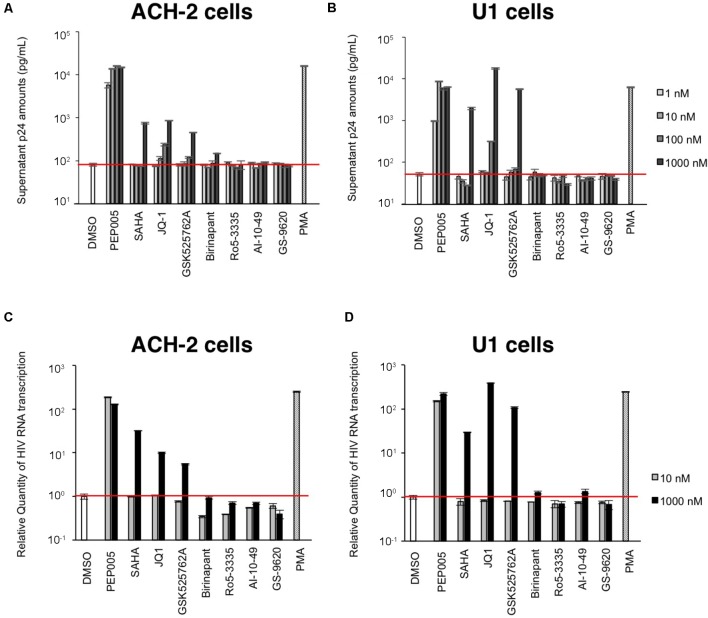
**(A,B)** The increase in viral production from ACH-2 cells **(A)** and U1 cells **(B)** was examined by supernatant p24 antigen levels on day 2. **(C,D)** Changes in the intracellular HIV-1 mRNA levels due to treatment with these drugs in ACH-2 cells **(C)** and U1 cells **(D)** after 24 h of incubation.

First, we examined the effect of the selected LRAs on latent HIV-1-infected cells. As shown in **Figure 1**, some LRAs tested in this study (PEP005, SAHA, JQ-1, and GSK525762A) increased viral production from ACH-2 cells and U1 cells, which was determined by the supernatant p24 antigen levels. Among them, PEP005 most potently reactivated and increased the supernatant HIV-1 p24 levels of ACH-2 cells; the increase was >100-fold when compared with that of unstimulated ACH-2 cells. Other LRAs (Ro5-3335, AI-10-49, and GS-9620) failed to increase the p24 levels from ACH-2 cells and U1 cells (**Figures 1A,B**) in our experiments. As most previous studies used the intracellular HIV-1 mRNA levels of the reactivated cells to evaluate the magnitude of reactivation by LRA (Bullen et al., 2014; Jiang et al., 2015; Laird et al., 2015), we too assessed the changes in the intracellular HIV-1 mRNA levels that occurred because of the treatment. We found that the reactivation profiles of LRAs were similar to those determined in the supernatant p24 levels (**Figures 1C,D**). We simultaneously investigated the effect of birinapant on the latent HIV-1-infected cells. Pache et al. (2015) previously reported that treatment with some Smac mimetics enhanced HIV-1 transcription (Pache et al., 2015). However, birinapant only had a minor effect on HIV-1 reactivation in the ACH-2 cells and U1 cells as determined by the supernatant p24 levels (**Figures 1A,B**) and intracellular HIV-1 mRNA in this study (**Figures 1C,D**). Altogether, it is considered that the ability of birinapant to reactivate latent HIV-1-infected cells is not as potent when used alone when compared with that of LRAs in these cells. We then tested the effects of birinapant on productive HIV-1 infection using MT-4 cells and HIV-1 NL4-3 (**Supplementary Figure S1**). An RT inhibitor (EFdA/MK-8591) (Nakata et al., 2007; Kawamoto et al., 2008) potently inhibited the replication of HIV-1 NL4-3, but PEP005 and birinapant had either little to no effect on viral replication. Moreover, they had no additive/synergistic antiviral effect when combined with EFdA (**Supplementary Figure S1**).

In addition, we examined the toxicity of the test drugs on a variety of cell lines. Most compounds such as PEP005, SAHA, and JQ-1 showed minor toxicity on all tested cell lines. However, some drugs had lower 50% cytotoxicity concentration (CC_50_) values for particular cell line(s). For example, AI-10-49 had either minor or no toxicity toward many cells but was toxic toward Jurkat cells (CC_50_: 1.1 μM). The Smac mimetic, birinapant, also showed toxicity toward A3.01 cells (CC_50_: 0.2 μM), but it only showed minor toxicity toward other tested cells (**Table 1**). This may be explained by the fact that the patterns of expression of various cellular factors are invariably different in each cell, thus, causing the differences in the toxicity profiles of each drug toward different cells.

**Table 1:** Cytotoxicity of tested LRAs and Smac mimetic.

Function/action	Compound	CC_50_ ± SD (μM), against							
		
		A3.01	ACH-2	Jurkat	J1.1	U937	U1	HL-60	OM10.1
PKC activator	PEP005	47 ± 9	19 ± 0.4	16 ± 3	44 ± 2	>100	>100	>100	84 ± 6
HDAC inhibitor	SAHA	>100	9 ± 1.2	90 ± 2	>100	>100	>100	>100	>100
BRD4 inhibitor	JQ-1	25 ± 0.3	22 ± 0.9	22 ± 0.7	29 ± 0.1	31 ± 0.8	38 ± 0.02	32 ± 0.8	21 ± 0.2
	GSK525762A	>100	>100	>100	>100	>100	>100	>100	>100
Smac mimetic	Birinapant	0.2 ± 0.08	>100	13 ± 0.1	48 ± 0.9	>100	>100	>100	53 ± 8
CBFb inhibitor	Ro5-3335	>100	>100	>100	>100	>100	>100	>100	>100
	AI-10-49	>100	41 ± 10	1.1 ± 0.07	>100	>100	>100	>100	>100
TLR7 agonist	GS-9620	>100	>100	23 ± 0.3	35 ± 1.3	>100	>100	>100	>100


*Values represent mean values (±1 SD) of two or three independent experiments.*

### Birinapant Suppresses HIV-1 Production From Latent Cells Reactivated by TNF-α or PEP005

**FIGURE 2:**
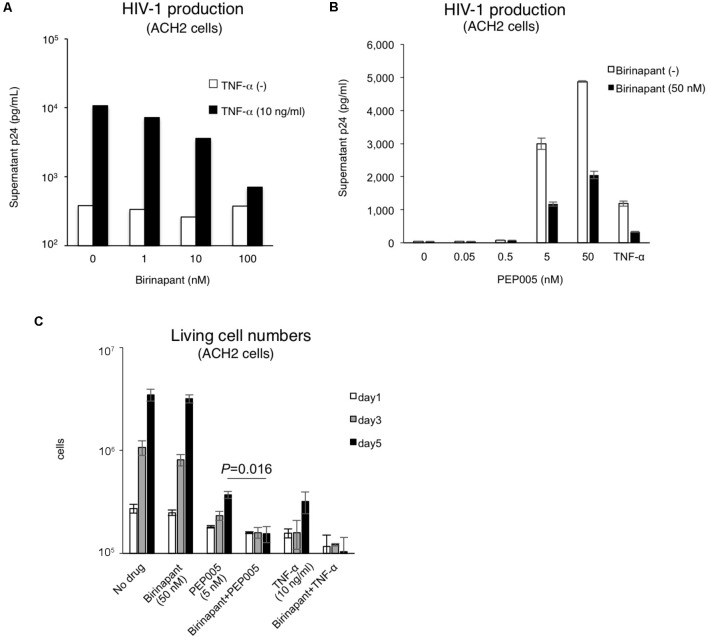
**(A)** Effect of birinapant when combined with TNF-α. The supernatant p24 levels with birinapant (1∼100 nM) in ACH-2 cell culture on day 2 are shown in white bars, and p24 values with birinapant when combined with TNF-α (10 ng/ml) are shown in black bars. **(B)** HIV-1 production from ACH-2 cells when reactivated by PEP005 (0.05∼50 nM) for 24 h. **(C)** Changes in the living cell numbers in the presence of drug(s) on days 1, 3, and 5.

As shown in **Figure 1**, birinapant only had a minor effect on HIV-1 reactivation when administered alone. Hence, we subsequently examined the effect of birinapant when combined with TNF-α or an LRA. The administration of birinapant (1∼100 nM) to ACH-2 cells failed to affect supernatant p24 antigen levels (**Figure 2A**). On the other hand, TNF-α (10 ng/ml) drastically increased the supernatant HIV-1 (p24) levels by >20-fold when compared with that of unstimulated cells. However, when the cells were treated with a different concentration of birinapant with TNF-α, the p24 levels in the supernatant decreased in a birinapant concentration-dependent manner (**Figure 2A**). Similar result was obtained when a different cell line, J1.1, was used (**Supplementary Figure S2**). Subsequently, the effect of PEP005 (one of the most potent LRAs) with birinapant was examined. As shown in **Figure 2B**, TNF-α and PEP005 (5 nM and higher) reactivated and increased the supernatant HIV-1 p24 levels obtained from ACH-2 cells after 24 h. However, similar to the results observed with TNF-α and birinapant, the supernatant p24 levels drastically decreased (∼40%) when the cells were simultaneously treated with birinapant (50 nM). Hence, we probed into the mechanism of how the combination reduced the viral production from the reactivated latent cells. As shown in **Figure 2C**, the treatment with birinapant (50 nM) failed to show cell toxicity in ACH-2 cells. On the other hand, 5 nM of PEP005 failed to kill the cells, but it strongly inhibited the replication of ACH-2 cells (3.70 × 10^5^/ml on day 5). Moreover, the number of living cells treated with PEP005 and birinapant was much less (1.55 × 10^5^/ml on day 5) than those treated with PEP005 alone (**Figure 2C**). We questioned if the combination of birinapant with PEP005 or TNF synergistically induced strong, non-specific cell toxicity, and, thus, we examined their toxicity using primary T-cells from a healthy donor. As shown in **Supplementary Tables S1**, **S2**, the combination of PEP005 and birinapant (both 10 nM to 1 μM) failed to enhance toxicity in uninfected cells. We also examined the toxicity of the combination in primary cells in HIV-1-infected patient (under cART treatment) (**Supplementary Figure S3**). Single treatment with birinapant (50 nM) slightly increased the ratio of dead cells (when determined by PI staining), but PEP005 (5 nM) alone and the combination (PEP005 + birinapant) failed to increase the number of dead cells (**Supplementary Figure S3**). These results suggest that the combination of PEP005 and birinapant with concentrations used in this study does not have non-specific cell toxicity. Altogether, it is considered that the strong toxicity observed in ACH-2 cells treated with the combination of PEP005 and birinapant suggests the presence of certain mechanisms by which latent HIV-1-infected cells are killed when the two are taken together.

### Combination of Birinapant and PEP005 Enhances Caspase-3 Signaling in HIV-1 Latent Cells

**FIGURE 3:**
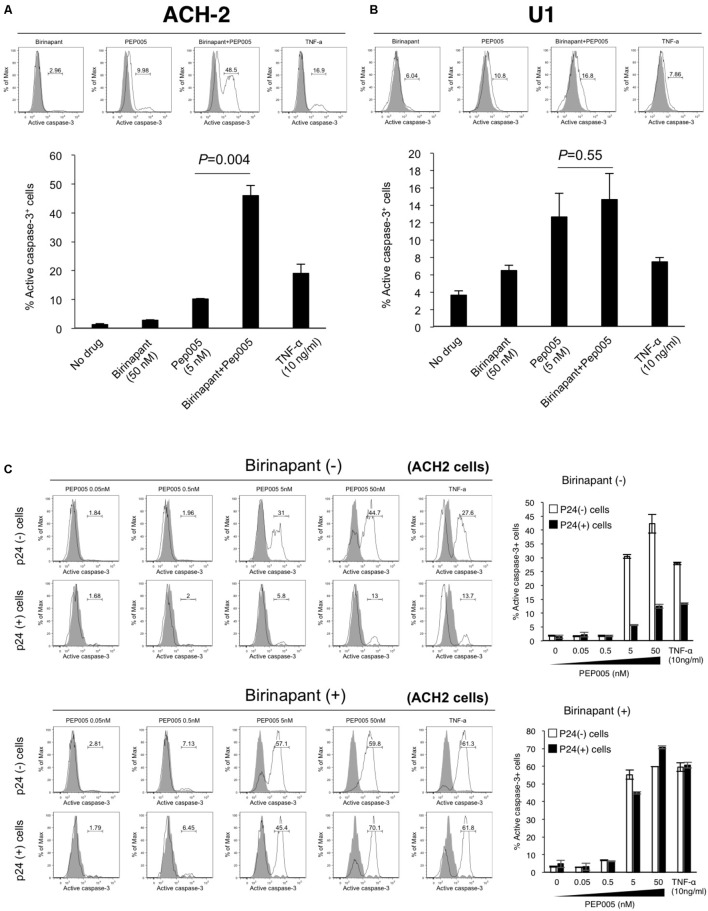
**(A,B)** ACH-2 **(A)** and U1 cells **(B)** were treated with birinapant (50 nM), PEP005 (5 nM), birinapant plus PEP005, TNF-α (10 ng/ml), and intracellular expression of caspase-3 was examined after 24 h of incubation. The percentage of active caspase-3 cells was determined by comparison with unstimulated cells. **(C)** ACH-2 cells after 24 h of PEP005 (0∼50 nM) treatment with or without birinapant (50 nM) were separately analyzed in p24^+^ and p24^-^ populations for caspase-3 expression.

If birinapant inhibits the effect of PEP005 (and TNF-α) as a reactivator of latency and, thus, reduces HIV-1 production, the combination should not be considered as a treatment option for HIV-1 reservoirs. However, if birinapant acts to reduce HIV-1 production through a certain mechanism, without affecting the LRA activity to “kick (shock)” latent cells, it can be a very interesting strategy. Hence, we hypothesized that the “survival” signal that causes HIV-1-DNA transcription, induced by TNF-α or PEP005, is converted by birinapant to the “death” signal that causes the activation of the cell apoptosis pathway. The latent HIV-1-infected cells (ACH-2 and U1 cells) were treated with the drug(s), and the intracellular expression of active caspase-3 was examined after 24 h of incubation (**Figure 3**). Single treatment with either birinapant or PEP005 was observed to have increased the expression of active caspase-3 in ACH-2 cells (2.96 and 9.98%, respectively), but the levels were drastically enhanced to 48.5% when they were combined. Latent monocyte-derived HIV-1-infected U1 cells responded more strongly to single treatment with PEP005 (10.8%), whereas the combined effect was less (*P* = 0.55) than that of ACH-2 cells (**Figures 3A,B**).

Next, we investigated whether the upregulation of active caspase-3 occurred in HIV-1 producing cells or in non-producing cells. Cells (ACH-2) after 24 h of drug treatment were separately analyzed in p24-positive (p24^+^) and p24-negative (p24^-^) populations (**Supplementary Figure S4** and **Figure 3C**). Interestingly, we found that PEP005 alone enhanced the active expression of caspase-3 in mostly p24^-^ cells (44.7 vs. 13.0% with 50 nM PEP005). However, the combination treatment with birinapant and PEP005 successfully enhanced the active expression of caspase-3 in both p24^-^ and p24^+^ cells (59.8 and 70.1%, respectively), especially in p24^+^ cells, the expression drastically increased when compared with the treatment without birinapant (13.0% without birinapant vs. 70.1% with birinapant) (**Figure 3C**). These results suggest that PEP005 (LRA) can induce the apoptosis signal but mostly only in HIV-1 non-producing cells. However, the combination of PEP005 and birinapant can enhance the caspase signal more strongly in both HIV-producing and non-producing cell populations.

### Immediate Killing of the Latent HIV-1-Infected Cells Before They Produce Viral Particles Helps to Decrease HIV-1 Viremia

**FIGURE 4:**
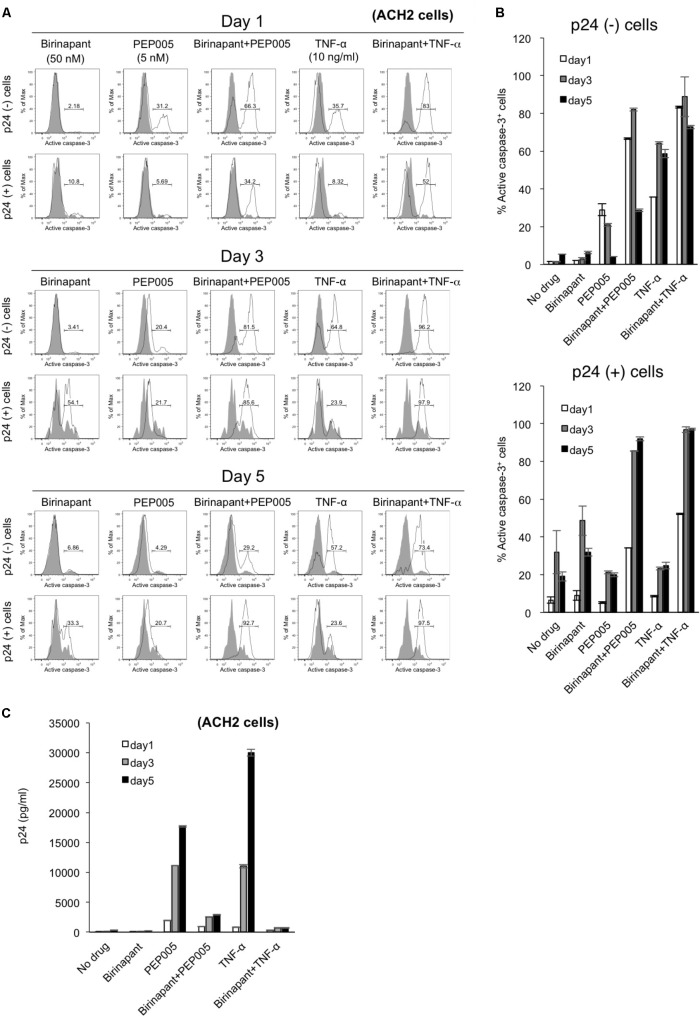
**(A)** ACH-2 cells were exposed to birinapant (50 nM), PEP005 (5 nM), birinapant plus PEP005, TNF-α (10 ng/ml), or birinapant plus TNF-α treatment and were cultured for 5 days, and data (FACS analysis) were collected at days 1, 3, and 5. **(B)** Expression of active caspase-3 in p24^+^/^-^ populations is shown. **(C)** Changes in the supernatant p24 antigen levels in the presence of drug(s) on days 1, 3, and 5.

To determine the fate of latent HIV-1-infected cells with upregulated caspase-3 expression, we conducted a time-course analysis with PEP005 and birinapant. ACH-2 cells were exposed to the drug(s), cultured for 5 days, and the comprehensive data (p24 expression and caspase expression) were collected on days 1, 3, and 5 (**Figure 4**). Changes in caspase-3 expression with each drug at each data point (**Figure 4A**) are summarized in **Figure 4B**. In a p24^-^ population in ACH-2 cells (considered as a latent cell population), PEP005 (single and birinapant-combined) rapidly induced the activation of caspase-3, reaching the highest level on days 1 or 3 (**Figures 4A,B**, upper graph). On the contrary, it took slightly longer for the p24^+^ population to turn caspase-3 positive (its peak level was observed on days 3 or 5). In addition, more importantly, PEP005 alone failed to activate caspase-3 in the p24^+^ population (∼20.7% on day 5) (**Figures 4A,B**, lower graph). However, the combination of PEP005 and birinapant induced the active expression of caspase-3 in >90% of the p24^+^ cells by day 5 (**Figures 4A,B**, lower graph), presumably resulting in the death of these cells, which was then followed by reduced viremia (supernatant p24) levels when compared with that of treatment with TNF-α only (**Figure 4C**). As shown in **Figure 2C**, it is notable that the number of living cells (including p24^+^ and p24^-^ ACH-2 cells) after combination treatment with PEP005 and birinapant (3.70 × 10^5^/ml) was 10.6% of the number seen in cells that undergo no treatment, suggesting a unique feature that only the combination of birinapant and PEP005 can induce caspase-3 upregulation in both p24^+^ and p24^-^ populations and can kill these cells. This is important to kill most of the latent HIV-1-infected ACH-2 cells with drastically reduced supernatant p24 values (**Figure 4C**).

### Combination Effect of Birinapant With PEP005 in HIV-1-Infected Primary CD4^+^ T-Cells From HIV-1-Infected Patients

**FIGURE 5:**
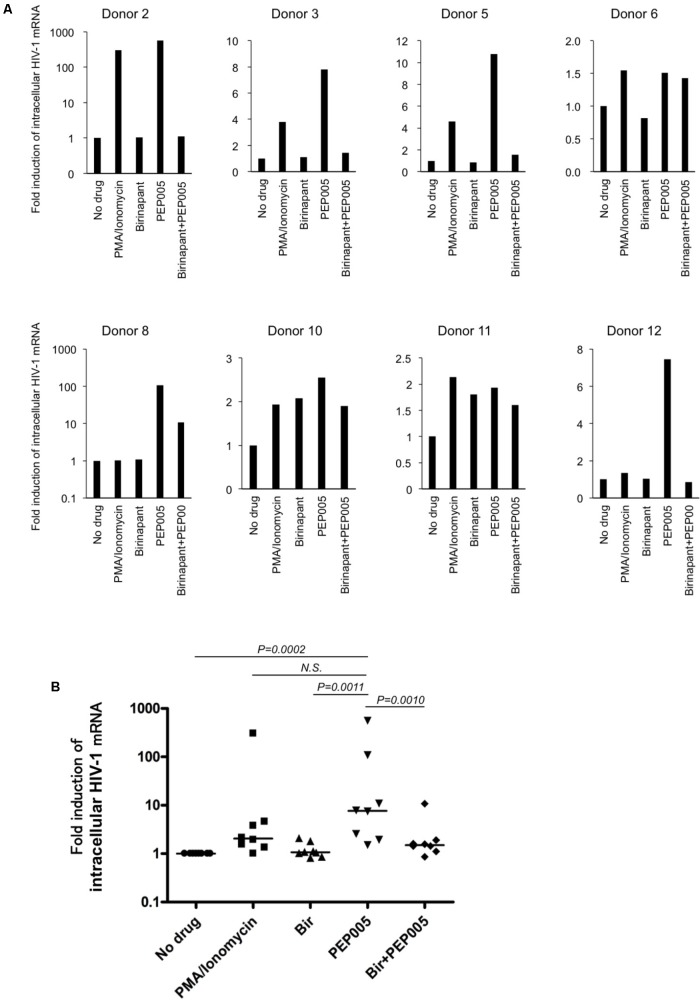
**(A)** Changes in the HIV-1 mRNA expression on primary CD4^+^ T-cells from HIV-1-infected patients in the presence of PEP005 and/or birinapant. Cells were incubated with/without drug(s) for 24 h and the levels of HIV-1 mRNA were compared. Eight patient samples reacted to PMA/Ionomycin and the combined effect of drugs (5 nM PEP005 and 50 nM birinapant) was examined. **(B)** All data (donors 2, 3, 5, 6, 8, 10, 11, and 12) for HIV-1 mRNA **(A)** was plotted and each drug treatment group was compared. Statistical significance was determined using Mann-Whitney U test, where *P*-value < 0.05 was considered to be significant.

Finally, we examined the effect of birinapant in combination with PEP005 in primary CD4^+^ T-cells from HIV-1-infected patients under cART in Japan (**Supplementary Table S3**). Primary cells from HIV-1-infected patients were treated with PEP005 and/or birinapant for 24 h, mRNA was extracted and the level of HIV-1 mRNA was measured. Similar to the results observed in the cell lines (**Figures 1C,D**), PEP005 treatment elevated the expression of HIV-1 mRNA in primary CD4^+^ cells in eight samples (**Figure 5A**). However, when PEP005 was added in combination with birinapant, HIV-1 mRNA expression decreased when compared to that seen with PEP005 alone in all cells. The single treatment with birinapant failed to change the HIV-1 mRNA expression in most samples, but cells from two donors (donors 10 and 11) reacted to birinapant and showed an increase in mRNA expression (**Figure 5A**). Overall, PEP005 (5 nM) exposure enhanced the expression of HIV-1 mRNA when compared to that without drug (*P* = 0.0002), and the addition of birinapant (50 nM) with PEP005 resulted in the decrease in mRNA expression when compared to the treatment with PEP005 alone (*P* = 0.001) (**Figure 5B**). This result suggests that the same trend was seen in the effect of birinapant with PEP005 between data obtained using cell lines and that obtained using primary cells from HIV patients.

## Discussion

**FIGURE 6:**
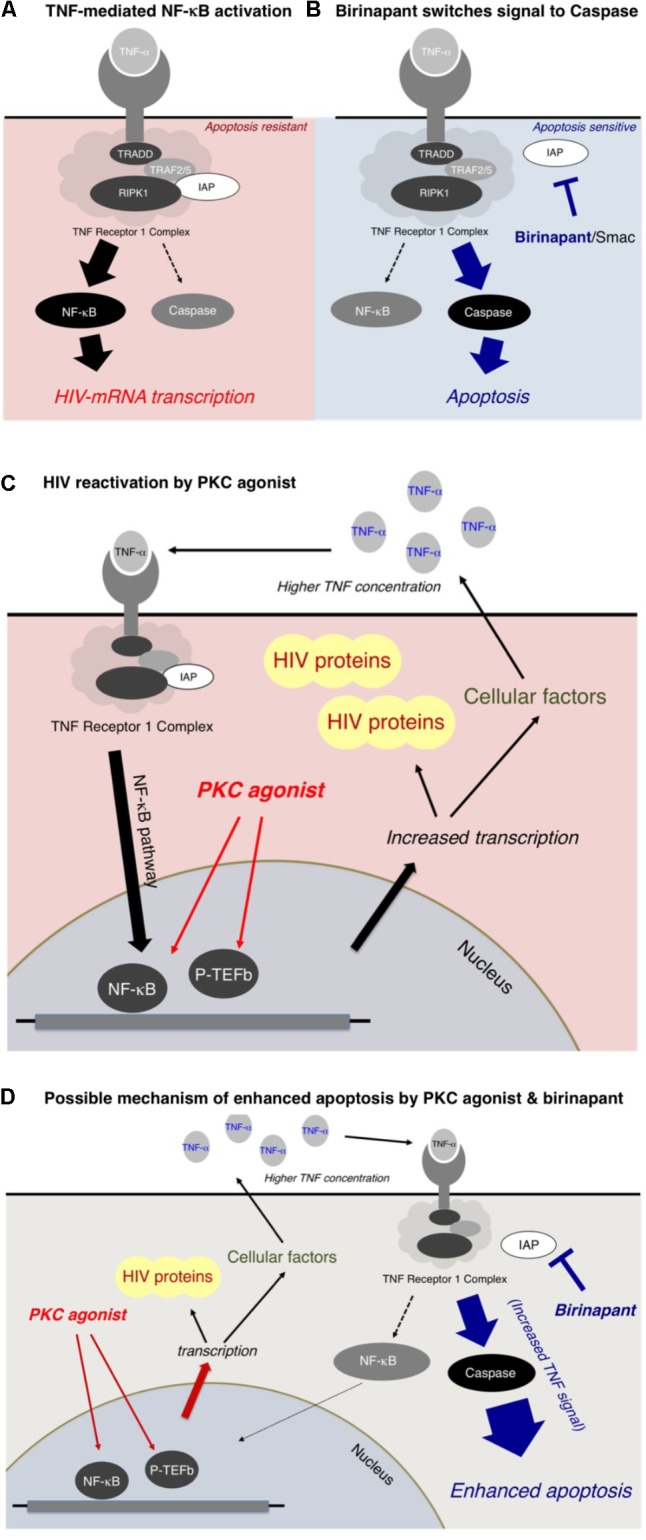
**(A,B)** Mechanism of enhanced apoptosis by birinapant through TNF signaling. **(C,D)** Possible mechanism of HIV-1 reactivation by PKC activators and apoptosis by birinapant.

In the present study, we reported that a Smac mimetic, birinapant, strengthened the apoptotic signal induced by LRAs such as PEP005 (a PKC activator). The possible mechanism of enhanced apoptosis by birinapant is summarized in **Figure 6**. **Figure 6A** illustrates the signal transduction induced by TNF-α (Allensworth et al., 2013; Badley et al., 2013; Bai et al., 2014; Condon et al., 2014; Darcis et al., 2017). The TNF receptor 1 complex receives a TNF-α signal and then transmits it inside the cell, triggering a cascade of events that includes the activation of NF-kB, which results in the increase in HIV-1 mRNA transcription. Cellular inhibitors of apoptosis are considered to increase the TNF-α-induced NF-kB activation rather than caspase activation (**Figure 6A**; Silke, 2011; Badley et al., 2013; Benetatos et al., 2014; Carter et al., 2014; Darcis et al., 2017). On the contrary, birinapant, an antagonist of cIAP, is considered to shift the signal from NF-κB activation to caspase activation (**Figure 6B**). The binding of birinapant to cIAP results in the decreased expression of cIAP in the cytoplasm (**Supplementary Figure S5**, lane 2), whereas the addition of either PEP005 or TNF-α induces the upregulation of cIAP-1 (and cIAP-2) (lane 3 and 5). However, when PEP005 or TNF-α were combined with birinapant (lane 4 and 6), the cIAP levels dropped to similar or lower levels as observed with birinapant alone (lane 2). This explains the mechanism by which these combinations effectively induce apoptosis without increasing the level of HIV-1 transcription.

As illustrated in **Figure 6C**, a PKC activator, PEP005, does not directly interact with the TNF receptor 1 complex, but it is considered to result in NF-κB activation through multiple pathways (**Figure 6C**). The PKC activator enhances the transcription of (1) HIV-1 mRNA and (2) mRNAs of multiple cellular factors, including TNF-α. Hence, the supernatant TNF-α level is elevated (data shown in **Supplementary Figure S6**), resulting in the activation of the NF-κB pathway, which causes further acceleration of HIV-1 reactivation (**Figure 6C**). However, if birinapant is added simultaneously with the PKC agonist, the increased TNF signal contributes to the caspase pathway instead of NF-κB activation, thus, resulting in the drastic enhancement of apoptosis (**Figure 6D**). Therefore, such combinations are considered to help reduce the viral production from reactivated latent cells by decreasing signaling via NF-κB while preserving/enhancing apoptosis of HIV-1 latent cells.

As shown in **Figure 5**, we showed that birinapant decreased the expression of HIV-1 mRNA in PEP005-treated primary CD4^+^ T-cells from HIV-1-carrying patients. It is anticipated that the use of CD4^+^ T-cells from ART-treated HIV-infected individuals is a powerful and informative approach to test LRAs under physiological conditions. In this study, we also used patient-derived CD4^+^ T-cells to determine the effect of birinapant and PEP005 (**Figure 5**). However, owing to the blood volume limitation from a patient in our facility, we were only able to use three million cells per well for the drug stimulation experiments. It is predicted that the peripheral blood reservoir cells from ART suppressed-HIV-infected individuals are quite few, and this possibly resulted in the weak response against drug stimulations and the experimental variability in **Figure 5**. As summarized in **Figure 6**, it is expected that birinapant shifts the signal through TNF-R to the apoptosis prone cells and reduces NF-kB activation in the primary cells (**Figure 6**). However, further experiments and analyses are necessary to know the precise effect of birinapant in primary CD4^+^ cells of HIV-1-infected patients when combined with LRA.

Birinapant is currently an investigational drug for a variety of cancers, and it is to be noted that the activity of birinapant to induce apoptosis can vary among different cell types, and cancer cells (or cell lines derived from cancer cells) are likely to be more sensitive to the drug (Fulda and Vucic, 2012; Bai et al., 2014). However, it is noteworthy that birinapant is also currently being investigated for its use in the treatment of chronic HBV infection (Ebert et al., 2015), and a phase II clinical trial is currently ongoing (Alonso et al., 2017). Regarding the utility of birinapant for HIV-1 infection, especially to cure HIV-1, Pache et al. (2015) reported that certain Smac mimetics reactivated latent HIV-1-infected cells and raised their HIV-1 mRNA expression (Pache et al., 2015). On the other hand, birinapant, which we have employed in this study, failed to induce strong reactivation of HIV-1 (**Figure 1**). Presumably each Smac mimetic has a different profile in induction of apoptosis or reactivation of HIV-1 latency, and birinapant is likely to induce apoptosis rather than HIV-1 reactivation. Further experiments are required to know the exact effect of Smac mimetics that includes the effect of birinapant on the reactivation of latent HIV-1 cells.

Several groups have investigated and reported that various small molecule compounds have the ability to reactivate latent HIV-1 (Bullen et al., 2014; Jiang et al., 2015; Laird et al., 2015; Pache et al., 2015). The increase in HIV-1 mRNA expression followed by the increased production of HIV-1 virions or HIV-1 related proteins results in the capture of the reactivated cells by the immune systems such as CTL and, thus, eliminating the reservoir cells. However, cell death (by viral cytopathicity) or apoptosis of the reactivated HIV-1 latent cells is regarded as another important mechanism to decrease HIV-1 latent reservoir cells. As summarized in an article by Badley et al., productive HIV-1 replication can be cytotoxic for the cells, but HIV-1 production does not always kill these cells, and the reason for this remains unknown. However, it is evident that certain conditions (e.g., p53 activation) or certain drugs (e.g., Bcl2 antagonist) can modulate the condition of the latent HIV-1-infected memory CD4^+^ T-cells to turn into apoptosis-sensitive cells (Badley et al., 2013). Moreover, Khan et al. reported that triggering apoptosis by addition of cytotoxic drugs such as anticancer agents in latently infected cells activated HIV replication in a caspase-dependent manner (Khan et al., 2015). It is, therefore, likely that Smac mimetics such as birinapant are a potential tool to prime latent cells toward an apoptosis-prone phenotype, especially when combined with certain types of LRAs. Thus, focusing on the ability of various drugs to induce apoptosis in latent HIV-1-infected cells could be very important. In fact, the combination of birinapant with an LRA (PEP005) successfully reactivated and eliminated the HIV latent cells. However, the production of infectious viruses was minimized, presumably because the drug combination successfully killed the cells before HIV-1 particles were secreted from the cells.

As a strategy to minimize/nullify secretion of HIV-1 particles from latent cells, Darcis et al. (2017) introduced a novel “lock” strategy in their review article, focusing on suppressing residual levels of HIV-1 transcription to lock the virus in a deep latency state to prevent viral reactivation (Darcis et al., 2017). In contrast, Tateishi et al. (2017) suggested a method in which the infecting virus is “locked-in” the cells followed by the apoptosis of the host cells with a certain drug (Tateishi et al., 2017). In the present study, we demonstrated the combined effect of birinapant with PEP005 on latent HIV-1-infected cells. However, further experiments are necessary to examine how birinapant interacts with other compounds that act as an LRA. For example, Yang et al. (2009) reported a compound (5HN) that reactivated latent HIV-1 without inducing global T-cell activation (Yang et al., 2009). Furthermore, a study by Dar et al. (2014) presented certain compounds that modulate HIV gene expression fluctuations that in turn enhance latent cell reactivation with conventional transcriptional activators (Dar et al., 2014). These compounds should be further analyzed in combination with birinapant for their effectiveness against latent HIV-infected cells. On the other hand, it was recently reported that mammalian target of rapamycin (mTOR) inhibitors suppressed HIV transcription both through the viral transactivator of transcription (Tat) and via Tat-independent mechanisms (Besnard et al., 2016). Laird et al. (2014) also reported other small molecules, cardiac glycosides that potently inhibit HIV-1 gene expression (Laird et al., 2014). Such compounds that suppress HIV transcription should also be considered as important candidates as counterparts of birinapant for combination treatment of latent HIV-reservoirs.

Thus, it is a promising strategy to use LRAs in combination with agents that induce cell apoptosis specifically in LRA-reactivated HIV-1-infected CD4^+^ T-cells, to eliminate the HIV-1 reservoir without the release of the HIV-1 viremia from the reactivated cells. However, it is unknown if the machinery works in primary HIV latent cells or *in vivo* reservoir cells. We conducted experiments mainly using ACH-2 and U1 cells. The activity of LRAs to induce HIV expression in ACH-2 and U1 cells are well correlated. However, they lack the expression of intact Tat protein, therefore, we conducted the same experiment using J1.1 cells, which have a *tat* gene, and we obtained similar results (**Supplementary Figure S2**). Further experiments and analyses using primary CD4^+^ T-cell-derived HIV latent reservoir models (Saleh et al., 2007) and animal models (i.e., HIV-infected humanized mice or simian immunodeficiency virus (SIV)-infected macaques) will also be necessary to predict whether the combination treatment can actually reduce the size of the reservoirs in humans. However, it should also be noted that the results of clinical trials in humans may not always reproduce what has been observed *in vitro*.

## Author Contributions

KeM, S-IH, KoM, and KT designed and performed the experiments. KY, HG, SO, and HM provided suggestions for the experimental design. KeM wrote the manuscript.

## Conflict of Interest Statement

The authors declare that the research was conducted in the absence of any commercial or financial relationships that could be construed as a potential conflict of interest. The reviewer KS and handling Editor declared their shared affiliation.

## Supplementary Material

FIGURE S1 The combined effect of an RT inhibitor (EFdA/MK-8591) (Nakata et al., 2007; Kawamoto et al., 2008; Maeda et al., 2014) and PKC activator (PEP005) on the replication of HIV-1 NL4-3 in MT-4 cells. MT-4 cells were infected with HIV-1 NL4-3 in the presence or absence of drug(s), cultured for 12 days, and the supernatant p24 levels were measured.

FIGURE S2 Birinapant suppresses virus production by combining with TNF-α in J1.1 cells. Cells were treated with birinapant with () or without () TNF-α (20 ng/mL). After 48 h incubation, culture supernatant was harvested, and viral RNA was extracted. Viral copy numbers in culture supernatant were measured by real-time RT-PCR.

FIGURE S3 The combined effect of birinapant (50 nM) and PEP005 (5 nM) on cytotoxicity in PBMCs obtained from HIV-1-infected patients. PBMCs from a cART-treated HIV-1 patient were cultured for 24 h in the presence or absence of drug(s), and the percentage of propidium iodide (PI)^+^ cells were measured by flow cytometric analysis.

FIGURE S4 The expression of intracellular HIV-p24 proteins in ACH-2 cells. ACH-2 cells were exposed to different concentrations of PEP005 in the presence (50 nM) or absence of birinapant and intracellular p24 level was examined after 24 h. Subsequently active caspase-3 expression in each population was examined and shown in **Figure 3C**.

FIGURE S5 Western blotting (WB) analysis for IAP family proteins in ACH-2 cells. Cells were exposed to birinapant (50 nM) in the presence or absence of PEP005 (5 nM), with TNF-α (10 ng/ml) co-treatment for 24 h, and cell lysates were used for WB analysis.

FIGURE S6 Elevation of supernatant TNF-α protein and mRNA levels in the presence of PEP005. **(A)** ACH-2 cells were exposed to PEP005, incubated for 24 h, supernatant was harvested, and TNF-α protein levels were measured using ELISA. **(B)** Changes in mRNA levels of TNF-α in PEP005-treated primary CD4^+^ T-cells in eight HIV-1 patients (**Supplementary Table S3**). Cells were harvested after 24 h incubation.

TABLE S1 CC_50_ values on PHA-stimulated PBMCs from healthy donor.

TABLE S2 Toxicity of PEP005 and birinapant in combination.

TABLE S3 Clinical characteristics of patients employed in this study.

## Abbreviations

CC_50_: 50% cytotoxic concentration
