# Supplementary figures and images for: Combination of a Latency-Reversing Agent With a Smac Mimetic Minimizes Secondary HIV-1 Infection in vitro
Source: Front Microbiol. 2018 Sep 19;9:2022. doi: 10.3389/fmicb.2018.02022 (PMC6156138; doi:10.3389/fmicb.2018.02022)

Figure S1.

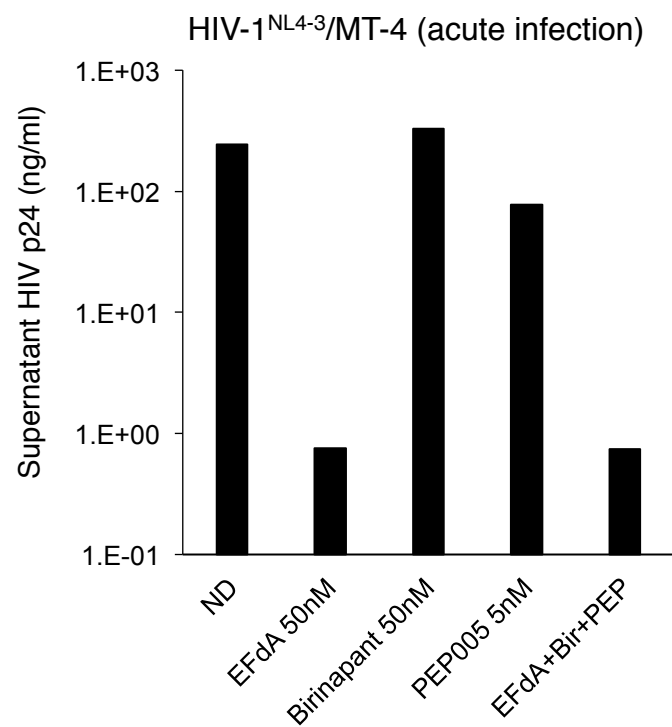

Supplement: FIGURE S1 — The combined effect of an RT inhibitor (EFdA/MK-8591) (Nakata et al., 2007; Kawamoto et al., 2008; Maeda et al., 2014) and PKC activator (PEP005) on the replication of HIV-1 NL4-3 in MT-4 cells. MT-4 cells were infected with HIV-1 NL4-3 in the presence or absence of drug(s), cultured for 12 days, and the supernatant p24 levels were measured. [file Data_Sheet_1.PDF]

Figure S2.

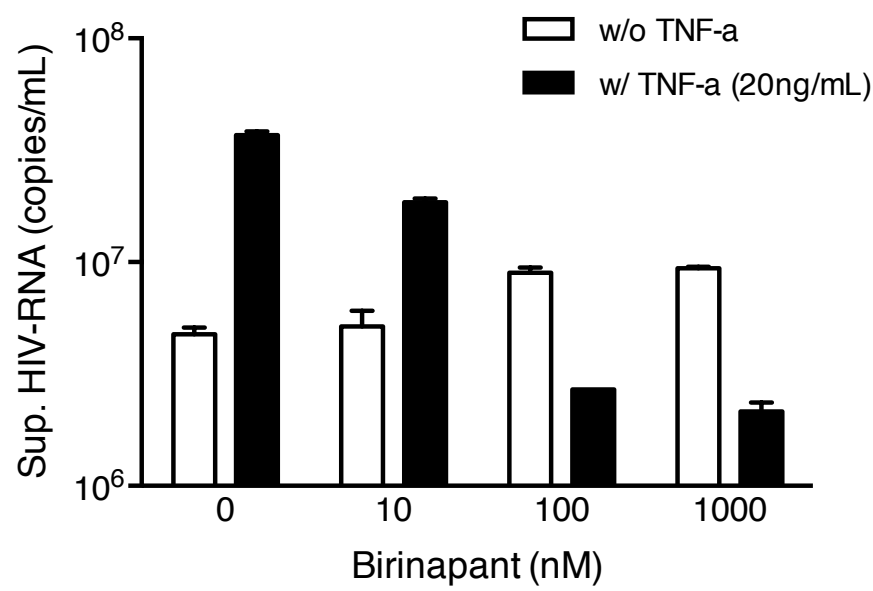

Supplement: FIGURE S2 — Birinapant suppresses virus production by combining with TNF-α in J1.1 cells. Cells were treated with birinapant with () or without () TNF-α (20 ng/mL). After 48 h incubation, culture supernatant was harvested, and viral RNA was extracted. Viral copy numbers in culture supernatant were measured by real-time RT-PCR. [file Data_Sheet_2.PDF]

Figure S3

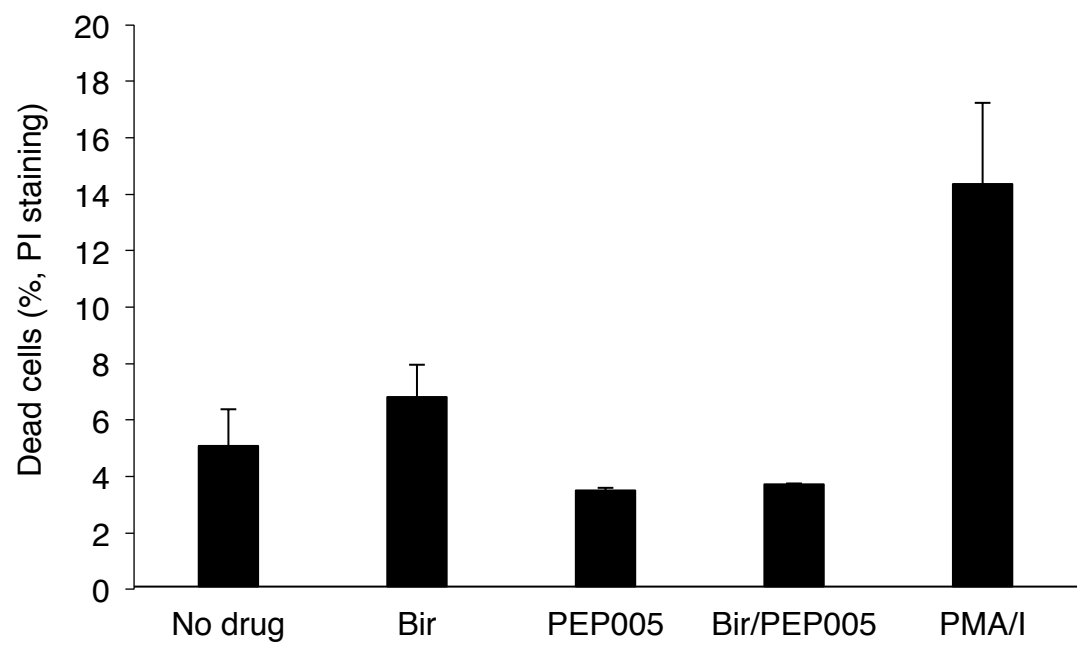

Supplement: FIGURE S3 — The combined effect of birinapant (50 nM) and PEP005 (5 nM) on cytotoxicity in PBMCs obtained from HIV-1-infected patients. PBMCs from a cART-treated HIV-1 patient were cultured for 24 h in the presence or absence of drug(s), and the percentage of propidium iodide (PI)+ cells were measured by flow cytometric analysis. [file Data_Sheet_3.PDF]

Figure S4.

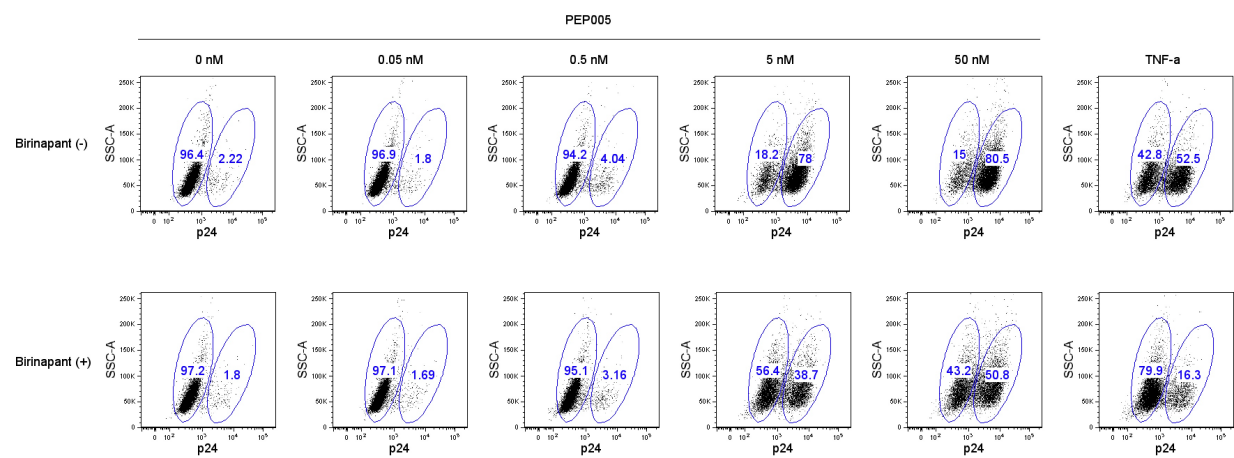

Supplement: FIGURE S4 — The expression of intracellular HIV-p24 proteins in ACH-2 cells. ACH-2 cells were exposed to different concentrations of PEP005 in the presence (50 nM) or absence of birinapant and intracellular p24 level was examined after 24 h. Subsequently active caspase-3 expression in each population was examined and shown in Figure 3C. [file Data_Sheet_4.PDF]

**Figure S5.**

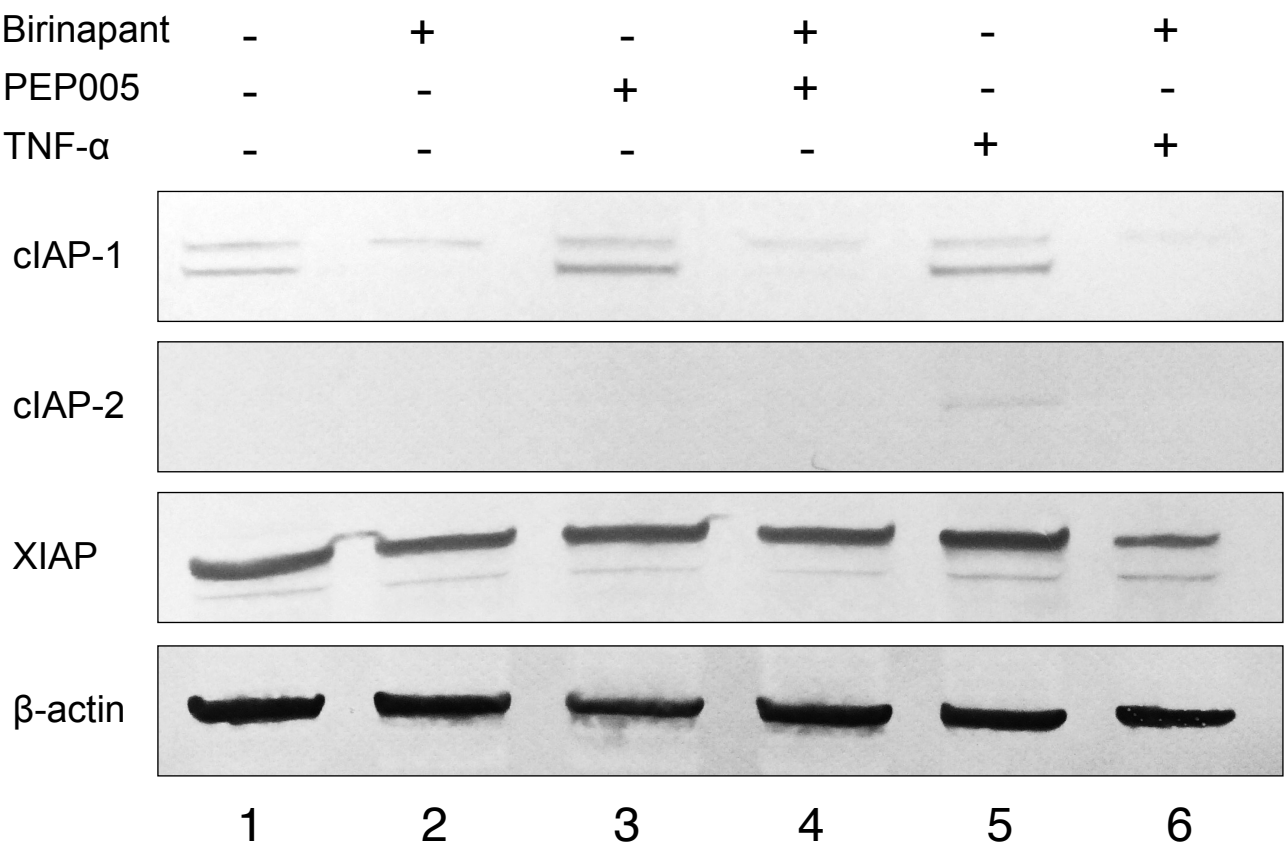

Supplement: FIGURE S5 — Western blotting (WB) analysis for IAP family proteins in ACH-2 cells. Cells were exposed to birinapant (50 nM) in the presence or absence of PEP005 (5 nM), with TNF-α (10 ng/ml) co-treatment for 24 h, and cell lysates were used for WB analysis. [file Data_Sheet_5.PDF]

**A**

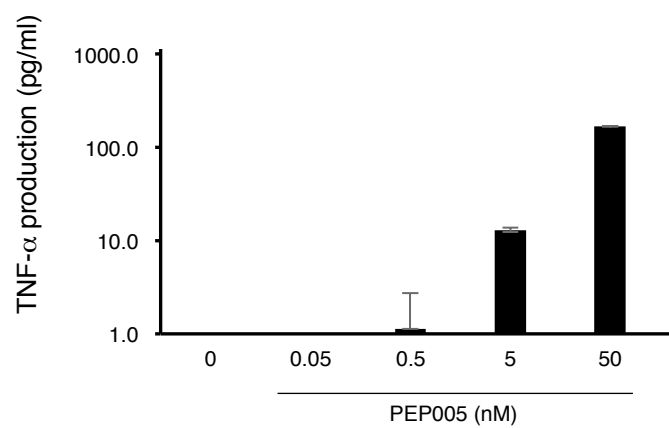

**B**

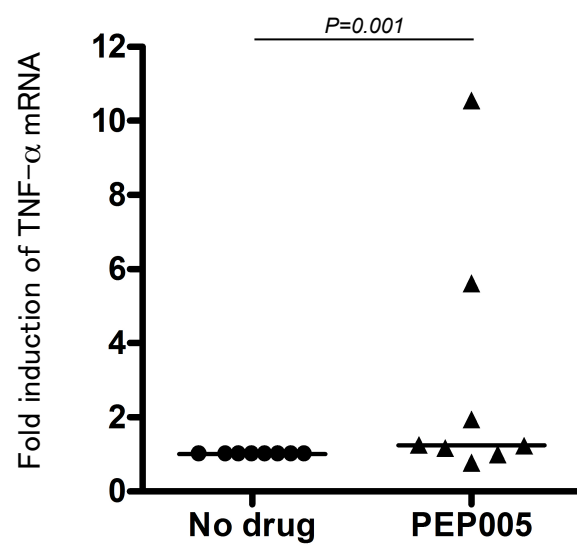

Supplement: FIGURE S6 — Elevation of supernatant TNF-α protein and mRNA levels in the presence of PEP005. (A) ACH-2 cells were exposed to PEP005, incubated for 24 h, supernatant was harvested, and TNF-α protein levels were measured using ELISA. (B) Changes in mRNA levels of TNF-α in PEP005-treated primary CD4+ T-cells in eight HIV-1 patients (Supplementary Table S3). Cells were harvested after 24 h incubation. [file Data_Sheet_6.PDF]
