# Supplementary material for: Combination of a Latency-Reversing Agent With a Smac Mimetic Minimizes Secondary HIV-1 Infection in vitro
Source: Front Microbiol. 2018 Sep 19;9:2022. doi: 10.3389/fmicb.2018.02022 (PMC6156138; doi:10.3389/fmicb.2018.02022)
Supplement: TABLE S1 — CC50 values on PHA-stimulated PBMCs from healthy donor. [file Table_1.pdf]

Table S1

Table S1. CC_{50} values on PHA-stimulated PBMCs from healthy donor

Agent	CC_{50} (μM) ± SD
PEP005	> 100
Birinapant	10.7 ± 0.2

PBMCs (2 x 10^6 cells/ml) were treated with birinapant or PEP005 and cultured for 3days. Cell viability was measured by WST-8 assay.
