# Supplementary material for: Combination of a Latency-Reversing Agent With a Smac Mimetic Minimizes Secondary HIV-1 Infection in vitro
Source: Front Microbiol. 2018 Sep 19;9:2022. doi: 10.3389/fmicb.2018.02022 (PMC6156138; doi:10.3389/fmicb.2018.02022)
Supplement: TABLE S2 — Toxicity of PEP005 and birinapant in combination. [file Table_2.pdf]

Table S2

Table S2. Toxicity of PEP005 and birinapant in combination

Agent (concentration)	% replication
PEP005 (10 nM) + birinapant (10 nM)	84.8 %
PEP005 (100 nM) + birinapant (100 nM)	80.8 %
PEP005 (1 μM) + birinapant (1 μM)	65.5 %
PEP005 (10 μM) + birinapant (10 μM)	50.0 %

PBMCs (2 x 10^6 cells/ml) were treated with birinapant (10 nM - 10 μM) and/or PEP005 (10 nM - 100 μM) and cultured for 3 days. Cell viability was measured by WST-8 assay.
