# Supplementary material for: Combination of a Latency-Reversing Agent With a Smac Mimetic Minimizes Secondary HIV-1 Infection in vitro
Source: Front Microbiol. 2018 Sep 19;9:2022. doi: 10.3389/fmicb.2018.02022 (PMC6156138; doi:10.3389/fmicb.2018.02022)
Supplement: TABLE S3 — Clinical characteristics of patients employed in this study. [file Table_3.pdf]

Table S3.

Clinical characteristics of patients employed in this study.

Patient	M/F	Age	VL* (copies/ml)	CD4 count* (cells/mm3)	cART** (at the time of study)	Therapy (years)	Plasma HIV-1 RNA <20 copies/ml since
2	M	46	45.5	526	ABC/3TC/ETR/RAL	20	2011
3	M	51	<20	807	DRV/COBI/DTG	17	2011
5	M	47	<20	464	FTC/TAF/NVP	18	2012
6	M	42	<20	474	FTC/TDF/RPV	12	2011
8	M	49	<20	509	FTC/TAF/COBI/EVG	6	2013
10	F	49	<20	762	FTC/TAF/DTG	20	2012
11	M	45	<20	600	FTC/TDF/RPV	10	2012
12	M	46	41.3	667	DRV/COBI/RAL	14	2012

*VL & CD4 count: at the time of the study
**3TC, lamivudine; ABC, abacavir; COBI, cobicistat; DRV, darunavir; ETR, etravirine; EVG, elvitegravir; DTG, dolutegravir; FTC, embricitabine; NVP, nevirapine; RAL, raltegravir; RPV, rilpivirine; TAF, tenofovir alafenamide fumarate; TDF, tenofovir disoproxil fumarate
